# Effect of psycho-social support by teachers on improving mental health and hope of adolescents in an earthquake-affected district in Nepal: A cluster randomized controlled trial

**DOI:** 10.1371/journal.pone.0223046

**Published:** 2019-10-01

**Authors:** Rolina Dhital, Akira Shibanuma, Moe Miyaguchi, Junko Kiriya, Masamine Jimba

**Affiliations:** Department of Community and Global Health, Graduate School of Medicine, The University of Tokyo, Tokyo, Japan; University of the Witwatersrand, SOUTH AFRICA

## Abstract

**Introduction:**

Adolescents can be prone to mental health problems such as post-traumatic stress disorder (PTSD) and depression following disasters. School teachers can provide timely psycho-social support that could instill hope and improve mental health among adolescents in a post-earthquake situation in a low-resource setting. This study examined the effect of training for school teachers on psycho-social support on adolescents’ mental health and hope in an earthquake affected district in Nepal.

**Methods:**

This cluster randomized controlled trial was conducted in 15 schools in Dhading, a severely affected district by the 2015 earthquake in Nepal. The schools were randomized, as a result, 8 were in the intervention group and 7 in the control group. A total of 1,220 adolescents were recruited at baseline of which 605 adolescents belonged to intervention group and 615 to control group. The follow-up rate at 6 months was 83%. This trial was registered with Clinicaltrials.gov with registration number NCT03387007.

**Results:**

The intervention did not show significant effects for PTSD symptoms (Intervention*time, β = 0.33, p = 0.536), depression symptoms (Intervention*time, β = 0.30, p = 0.249), and hope (Intervention*time, β = -0.23, p = 0.588), among the adolescents at 6 months follow-up.

**Conclusion:**

The intervention did not improve mental health symptoms and hope among adolescents at 6 months follow-up. More focused and longer training could be necessary to address mental health among adolescents affected by earthquake. Additionally, longer follow-up could be necessary to assess the changes taking place over time.

**Trial registration:**

ClinicalTrials.gov NCT03387007.

## Introduction

Globally, 10–20% of children and adolescents are affected by mental health problems [1]. Adolescence is a period between 10 and 19 years of age when major physical, psychological and behavioral changes take place [2]. Many adolescents become prone to mental health problems as they transition from childhood to adulthood. Around 50% of the mental health problems are believed to start by the age 14 [3]. However, most of the problems in adolescents often go unnoticed, particularly in low-resource settings [4].

Low-resource settings that are disaster-prone often face additional challenges to addressing mental health [5]. Lack of access to mental health care services can compound the problems faced by adolescents in disaster settings [6]. Disaster can take many lives and destroy homes or whole communities, causing serious physical and psychological injuries [7]. The psychological injuries can lead to mental health problems such as post-traumatic stress disorder (PTSD) and depression [8].

PTSD is the most commonly identified mental health problem in the aftermath of an earthquake [9]. It is a condition that can develop in people who have experienced unpleasant events [10]. It can occur due to either a single traumatic event such as accident or exposure to repeated trauma such as a community violence [10]. The symptoms usually begin within three months of the traumatic incident but can also begin many years later [11]. PTSD symptoms can last for a minimum of one month and may remain for many years if left untreated [12]. The symptoms of PTSD in adolescents could range from fear, bad dreams, and disturbing thoughts to anger and aggression [13]. However, not all adolescents who have experienced difficulties or trauma would suffer from mental health problems such as PTSD [14].

Depression is also a common mental health problem identified following an earthquake [9]. It is a common mood disorder that can begin in early childhood [4]. Unlike PTSD, it is not always related to a traumatic event. Various factors can contribute to it such as environment, family, friends, school, and community [15]. Depression is diagnosed when the symptoms last for a minimum of two weeks [10]. Symptoms of depression in adolescents include sadness, lack of sleep, crying, and emotional numbing [16, 17].

However, timely support from caring and responsible adults such as teachers can instill hope in adolescents, which could help them overcome difficulties [18]. The concept of hope includes the beliefs in one’s potential to achieve a goal and to initiate and sustain the way towards achieving the goal [19]. Hope is directed towards positive future expectations and working towards them [19]. Hope can help adolescents shape their future positively as they transition to adulthood [19, 20].

School is an important community setting to promote mental health and hope for adolescents [21]. Systematic reviews and meta-analyses on school-based psycho-social interventions for adolescents after natural disasters such as an earthquake have suggested positive findings [22, 23]. However, most of the psycho-social support interventions for children and adolescents in the previous studies were provided by health providers or psychologists [22, 23]. Though the psycho-social support provided by the health care providers are considered to be effective, such intervention is not always feasible in low-resource settings [23]. Most low- and middle-income countries (LMICs) are likely to continue facing shortage of health professionals, and the shortage of mental health professionals is severer [24].

School teachers could be more readily available and have a positive influence for children and adolescents [21]. Teachers can help students to believe in themselves and achieve their goals [18]. However, the evidence remains scarce on the effectiveness of teacher-mediated school based interventions on improving mental health outcomes. Only 4 teacher-mediated intervention studies were published between 2000 and 2017, which had focused on teachers after natural disasters [25–28].

The identified intervention studies were conducted in China [25], Turkey [26, 27], and Sri Lanka [28]. All the four studies indicated improved mental health symptoms among the adolescents after the intervention. However, the nature and the duration of the interventions were different in these studies. The study from China was a randomized controlled trial (RCT) conducted 12 months after the 2008 Sichuan earthquake that had focused on calligraphy treatment of 30 days and it showed improved PTSD symptoms after the intervention [25]. The study from Turkey was a pre and post intervention study conducted 4 to 5 months after the 1999 earthquake that was focused on training the school authorities and teachers on classroom-based school reactivation program with two follow-up studies [26, 27]. The study also showed significantly improved PTSD symptoms among the students [26, 27]. The study from Sri Lanka was a quasi-cluster RCT (cRCT) conducted 2 years after the Tsunami in 2006 that had focused on training for teachers through a program called ‘ERASE-Stress (Enhancing resiliency among students experiencing–Stress) which comprised of classroom-based interventions that helped children to cope with trauma and showed improved results for PTSD symptoms, depression symptoms, and hope [28]. Teacher-mediated interventions could be also sustainable and feasible in low-resource post-disaster settings such as in Nepal.

Nepal is a natural disaster-prone and low-income country where adolescents comprise 11.8% of the total population [2]. The country also ranks 11^th^ for earthquake risks worldwide [29]. In April of 2015, Nepal faced a devastating earthquake of 7.8 Magnitude followed by numerous aftershocks. The earthquake killed over 8,000 people, and over 6,000 were severely injured [30]. Over 400,000 homes and 16,000 schools were damaged across the country [30].

For disaster-prone settings such as in Nepal, evidence for the effect of school-based psycho-social support interventions for adolescents remains inadequate. We conducted this study to examine the effect of school teachers’ psycho-social support intervention on PTSD symptoms, depression symptoms, and hope among adolescents in earthquake-affected districts in Nepal.

## Methods

### Study design and settings

This study is focused on one of the two districts included in a larger cRCT. The original study was conducted in Dhading and Myagdi districts of Nepal. Initially, when we designed this study, we intended to have another district (Myagdi) which was one of the least affected districts by the earthquake as a control district. However, the district was affected by a flood and landslide during our study period which affected the lives of many people including the adolescents in the district. Thus, it was no longer served as a control district for this study. Moreover, each district had its own distinct characteristics and its own set of intervention and control groups that were independent of each other. Thus, this study presents the findings of Dhading district only.

Dhading is a severely affected district by the earthquake in 2015 [31]. Following the earthquake, 70% of 236 schools were damaged, and around 700,000 students were affected in Dhading [32]. The district had only one municipality at the time of data collection. The municipality had 15 government secondary schools which were included as clusters in this study.

### Study participants

School-going adolescents from grades six to eight from the selected schools were the participants of this study. Adolescents with a minimum attendance of 80% in the schools and without any known diagnosis of mental health problems were considered eligible to participate in this study. We did not include the adolescents with 20% and lower attendance rate in this study because our intervention aimed at training teachers to provide psycho-social support through daily classroom activities. Including the measurement of the students with low attendance rates could have confounded our findings.

The students were selected through simple random sampling from the total list of student identification (ID) numbers for each grade. Attempts were made to generate 100 random ID numbers for each school. The average number of students in each grade was around 40 to 50, and the average daily attendance rate was around 75 to 80% in each school.

### Pairing of schools and randomization

The 15 schools were divided into seven pairs, with one unpaired school. The pairing was done for schools by the district education officer (DEO) considering similar characteristics such as geographical location, school infrastructure, and number of students.

The names of all schools were written in separate pieces of paper and folded into opaque envelops. Each pair of schools were grouped together and one school from each pair was randomly assigned to either group A or B. The groups were then randomly assigned as the intervention or control group through concealed allocation by the DEO. The unpaired school was also randomly assigned to either group through the methods mentioned above. As a result, the intervention group had eight schools, and the control group had seven schools.

The schools in the intervention group received the training for school teachers on psycho-social support for adolescents. The schools in the control group did not receive any training on psycho-social support. Blinding was not done for the intervention because all schools were required to be informed about the intervention.

### Intervention

The Inter-Agency Standing Committee (IASC) has stated that the mental health and psycho-social support should be multilayered with the type of intervention directed and the target population for each layer being different [33]. The IASC guideline on mental health and psycho-social support in humanitarian settings suggests more generalized interventions for a wider population at the bottom and more specialized intervention for the smaller population at the top. The first layer of intervention at the bottom is basic service and security targeting the entire population affected by the disaster. The second layer focusses on the subpopulation who could uphold their mental health and psycho-social well-being with timely psycho-social support [33]. The third layer towards the top is targeted at a smaller group of people who would require more focused interventions by trained and supervised workers such as psychological first aids and basic mental health care provided at primary health care level [33]. The fourth and the topmost layer is specialized services which include a subgroup of a population who face significant difficulties requiring psychiatric care and psychological support provided by specialists [33]. The intervention in this study was a teacher-mediated school-based intervention which falls under the second layer of intervention as outlined in IASC guideline [33].

The intervention of this study was conducted on September 2, 2016 in Dhading. We adapted the training guidelines on psycho-social support for education in emergencies prepared by United Nations Relief and Works Agency (UNRWA) [34]. We consulted the Department of Psychiatry and Mental Health at Tribhuwan University Teaching Hospital in Kathmandu, Nepal, to refine the training manual further. The department nominated an experienced clinical psychologist who had worked closely with the children and adolescents for the training in Nepal. The clinical psychologist provided two days of training on psycho-social support for the school teachers. The training comprised eight sessions in total with one to two hours for each session [34]. The training was aimed at enabling the teachers to apply the different components of psycho-social support in their daily classroom activities.

For the intervention group, the school principals from each school nominated two teachers to participate in the training. In total, 16 teachers participated, and these were teaching the adolescents from grades six to eight. The sessions covered the following topics:

1. Key concepts and principles of psycho-social support: According to UNWRA’s training manual, the purpose of this topic was to introduce key concepts and principles of psycho-social support to the teachers [34]. The training activities included matching key concepts with their definitions to assess the knowledge of the teachers. The session highlighted that it is not necessary for the teachers to be health professionals to be able to help the adolescents cope and overcome difficult life situations.
2. How children react to a crisis situation: The purpose of this topic was to explore the feelings and reactions of the adolescents to the difficult situations [34]. The session highlighted that adolescents and children can cope better in presence of responsible and caring adults such as teachers. The teachers can help create a calm and stable surrounding despite the difficulties. The training also addressed that majority of the adolescents could recover if basic psycho-social support is provided through classroom activities and recreational activities [34].
3. The role of teachers in promoting psycho-social wellbeing: The purpose of this topic was to facilitate the teachers to understand how they could find ways to promote psycho-social well-being of the students through daily classroom activities. It focused on subtopics such as how the teachers could promote a sense of security, a sense of identity, self-esteem, and hope among the adolescents even in crises [34].
4. How to discuss a crisis with children: The purpose of the topic was to facilitate teachers to understand and practice appropriate ways of communicating with the adolescents. The session highlighted that adolescents and children need factual information. It also highlighted the ways for teachers to start a conversation with the adolescents to help them overcome the feelings of guilt. Moreover, it highlighted the need to respect the privacy of the adolescents and not disclose the traumatic events in their lives in the public such as in front of the class. The session encouraged the teachers to promote healthy discussions among the adolescents while respecting their confidentiality [34].
5. Activities for improved learning and recovery: The purpose of this session was to facilitate the teachers to promote a creative platform and help students cope with the help of non-formal recreational activities in the school. The session highlighted that such session could help the adolescents recover traumatic experiences and regain their confidence [34].
6. How to manage challenging behavior in the classroom: This session intended to help the teachers explore positive approaches to handle challenging behavior in the classroom, and help such students through proper psycho-social support. It also facilitated the teachers to understand the reasons behind their actions and find positive non-threatening solutions for such adolescents [34].
7. Identifying and assisting children who may need more advanced support: The purpose of this session was to facilitate the teachers to recognize the symptoms and refer the ones in need to health centers for treatment. The session also highlighted the different layers of psycho-social support. The trainer encouraged the teachers to share their experiences and observations of the students and discuss on how they dealt with the situations. The trainer also encouraged them to share their future plans on how they intend to address such situations after the training [34].
8. Teachers’ wellbeing: This session highlighted the importance for teachers to look after their own emotional and mental well-being. The session facilitated the teachers to address their own stress and helped them find ways to handle their stress. The trainer encouraged the teachers to identify and discuss about their plans to handle their stress in the future [34].

The research team interacted with the teachers at 6 months follow-up through focus group discussions (FGD) to understand their perspectives on the usefulness of the training and the activities they conducted after the training. Majority of the teachers suggested that the training was useful, they were able to recall the components of the training, and suggested that they had been implementing the activities on a daily basis.

For the control group, the teachers for grades six to eight did not receive any training on psycho-social support. Instead, the school teachers for higher grades received training on nutrition as part of a separate study.

### Sample size

We calculated the minimum required sample size using the formula to detect a difference between two means [35]. The sample size was calculated based on the mean values of Child PTSD Symptom Scale (CPSS) from a similar intervention study conducted in Nepal [17]. The minimum required sample size for this study was calculated to be 498 for each group for independent sample t-test with a power of 80% and 5% level of significance with the two-sided alpha level. Thus, the total minimum required sample size was calculated to be 996.

**Fig 1:**
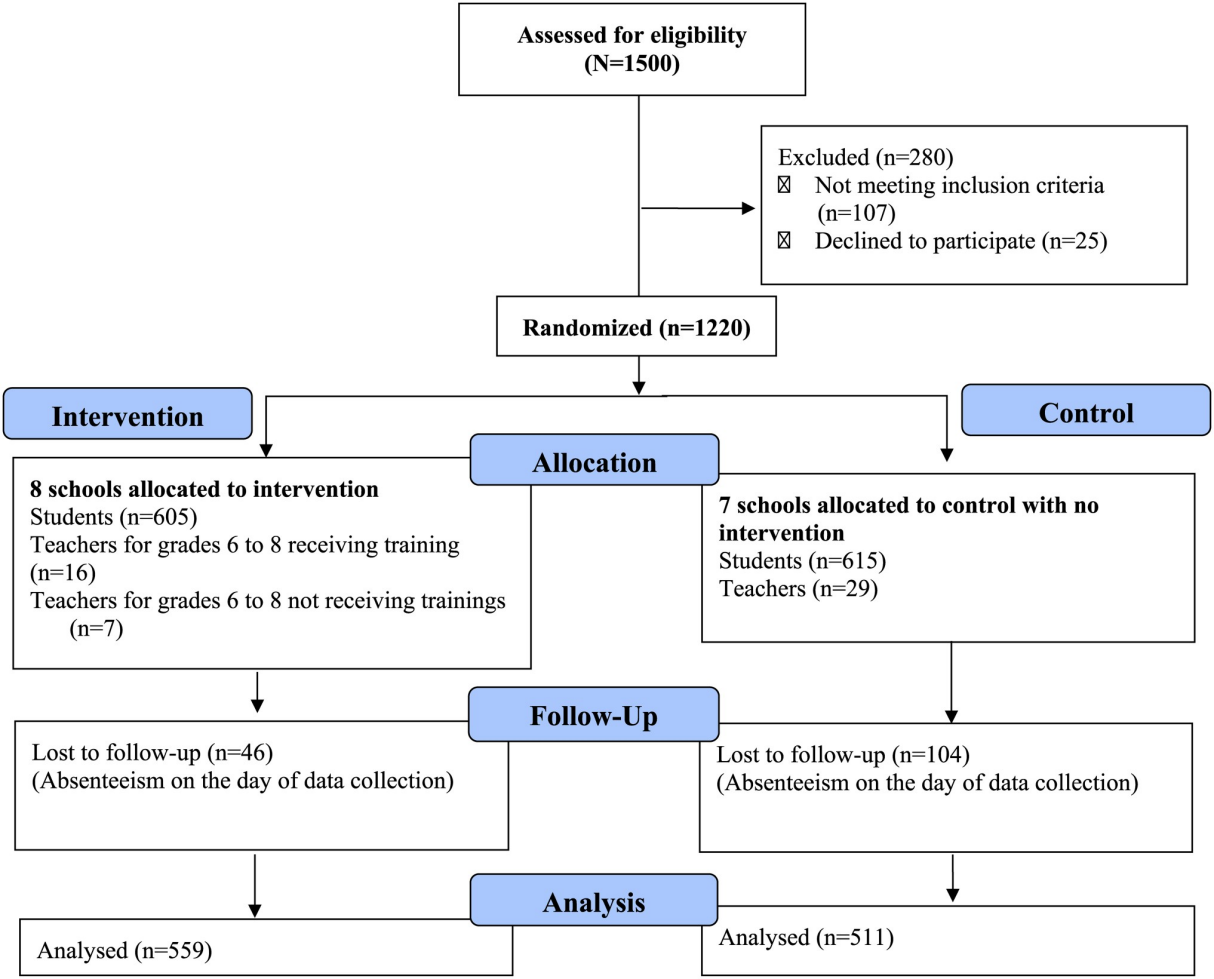
CONSORT flow chart.

Considering loss to follow-up, the sample size was extrapolated. A total of 1500 adolescents were screened for eligibility with an attempt to collect 100 students from each school. After exclusion, a total of 1220 adolescents were recruited in this study, 1070 of which were available for the follow-up. (Fig 1).

### Measures and instruments

The outcomes for this study were PTSD symptoms, depression symptoms, and hope among the adolescents. We used validated tools to measure the symptoms scores which are suggestive that the adolescents may have the symptoms but are not the definite diagnosis for the mental health conditions among the adolescents.

PTSD symptoms were measured using CPSS, which is a 17-item measure for children and adolescents [36]. The minimum score for this scale is 0, and maximum score is 52 with higher scores indicating severer symptoms for PTSD [36]. The scale has been validated in Nepal [16]. The scale includes questions related to re-experiencing, avoidance and hyperarousal. The scale included questions such as, “having upsetting thoughts or images about the event that came into your head when you didn’t want them to” [36].

Depression symptoms were measured using the Depression Self-Rating Scale, which is an 18-item self-report measure for children and adolescents [37]. The scores range from 0 to 36 with higher scores indicating severer symptoms for depression. The scale has also been validated in Nepal [16]. The questions on depression symptoms included emotional symptoms such as “I feel so sad I can hardly stand it” and physical symptoms such as “I feel tummy ache” [37].

Hope was measured using the Children’s Hope Scale (CHS), which is a six-item scale for children and adolescents [19]. The questions included the adolescents’ perspectives on belief in finding solutions to overcome difficulties such as, “when I have a problem I come up with lots of ways to solve it” and positive outlook towards future such as, “I think the things I have done will help me in the future” [19]. This scale was used in a previous study in Nepal [17]. The scores range from 1 to 36 with higher scores indicating better hope [19].

We included sociodemographic variables such as age, gender, and family size of the adolescents. We selected these variables based on a similar study [17].

### Data collection

The baseline data were collected between August 22, 2016 and August 31, 2016. The follow-up data were collected between February 1, 2017 and February 10, 2017. The research assistants were trained on data collection and ethical procedures. Following the training, they provided instructions and assisted the adolescents to complete the self-administered questionnaires in classrooms during regular school hours at both baseline and follow-up. The research assistants were available throughout the process to resolve any problem or explain questions to the adolescents. The adolescents took around 30 to 45 minutes to complete the questionnaire.

According to the original protocol, this study had aimed at collecting data at 3 months follow up after the intervention. However, the 3 month follow up was cancelled at the time of data collection due to feasibility problems.

### Ethical considerations

Approval for this study was obtained from The Research Ethics Committees of the Graduate School of Medicine at the University of Tokyo and Nepal Health Research Council. Permission to collect data from the selected schools were also obtained from the district education offices (DEO) of Dhading. The DEO in Dhading coordinated with the schools for this study. Permission was also obtained from the school principals for the adolescents to participate in the study within school hours. Written informed consent were provided by the adolescents who agreed to participate in the study and their guardians. The participation was voluntary and their confidentiality was maintained.

This study was registered retrospectively in ClinicalTrials.gov with the number NCT03387007. This study is a school based intervention that focused on training teachers and did not involve the direct experiment on the adolescents who were the main study participants. The researchers were not aware of the suitable registry for such community based cRCT conducted in Nepal prior to data collection. However, the authors confirm that all ongoing and related trials will be registered prospectively in the future.

### Data analysis

Data were analyzed using chi-squared tests, independent sample t-tests, and multivariable analysis using a generalized estimating equations (GEE) model. Chi-squared tests and independent sample t-tests were applied to assess the difference in the general characteristics between the intervention and control groups at baseline and follow-up. GEE models were used to examine the effect of teachers’ training on PTSD and depression symptoms and hope among the adolescents. The cluster effects were controlled for all the school in the GEE model. Other effects controlled in the GEE model included time, intervention, age, gender, and family size. We have used interaction effect in the GEE model to estimate the effect of the intervention under difference-in-differences method [38]. PASW Statistics 18.0 (SPSS Inc., Chicago, Illinois, USA) was used for all statistical analyses, and a *p* value of less than 0.05 was used as the significance level for all analytical procedures.

## Results

### Baseline characteristics of the adolescents

Out of 1,220 adolescents, 605 were in the intervention group and 615 in the control group. The mean age was almost 13 years in both groups. No significant difference was identified in gender between the intervention and control groups. The mean family size was almost five in both groups. (Table 1).

**Table 1:** Baseline characteristics of the adolescents.

	Intervention (n = 605)	Control (n = 615)	p-value
Variable	mean (SD)	mean (SD)	
**Age**^a^	12.9 (1.3)	12.9 (1.4)	0.351
**Gender**^b^**, n (%)**			
Male	289 (51.0)	278 (49.0)	0.369
Female	316 (48.4)	337 (51.6)	
**Family size**^a^	5.0 (1.7)	4.8 (1.6)	0.033

^a^Independent sample t-test

^b^Chi-squared test

### Differences in PTSD symptoms, depression symptoms and hope among adolescents

Table 2 demonstrates the differences in mean scores for PTSD symptoms, depression symptoms, and hope between the intervention and control groups in Dhading.

**Table 2:** PTSD symptoms, depression symptoms and hope in control and intervention groups^a^.

	Intervention mean (SD)	Control mean (SD)	p-value
**PTSD symptoms**			
Baseline	16.4 (7.9)	17.4 (8.1)	0.025
Month 6	16.4 (7.5)	16.8 (8.0)	0.477
**Depression symptoms**			
Baseline	12.7 (3.6)	13.2 (3.6)	0.037
Month 6	13.1 (3.8)	13.0 (3.7)	0.801
**Hope**			
Baseline	22.2 (6.1)	21.9 (6.1)	0.416
Month 6	22.3 (6.0)	22.3 (6.3)	0.856

^a^Refer to Fig 1 for ‘n’ at baseline and follow for each group.

No major differences were identified for PTSD symptoms between the intervention and control groups. However, the mean score of PTSD symptoms among adolescents at baseline in the intervention group (mean 16.4, SD 7.9) was slightly but significantly lower than those in the control group (mean 17.4, SD 8.1, p = 0.025). No significant difference was determined for PTSD symptoms at follow-up between the intervention and control groups.

The mean score for depression symptoms at baseline was also slightly but significantly lower among the adolescents in the intervention group (mean 12.7, SD 3.6) than those in the control group (mean 13.2, SD 3.6, p = 0.037). At follow-up, no significant difference was determined for depression symptoms between the groups.

No significant difference was determined for hope at baseline between the intervention and control groups. No significant difference was determined for hope between the groups at follow-up as well.

### Effect of the intervention

The intervention did not show evidence of a significant effect on PTSD symptoms at follow-up. PTSD symptoms did not change significantly over time and no significant difference was observed between the schools in intervention and control groups. (Table 3).

**Table 3:** GEE analyses; effect of intervention on PTSD symptom scores among adolescents^¶^.

	N = 2199^c^		
Variable	*β*	(95% CI)	p-value
Intervention*Time^a^	0.33	(-0.71, 1.37)	0.536
**Time**			
Baseline^b^			
Month 6	-0.69	(-1.44, 0.06)	0.073
**School**			
Control^b^			
Intervention	-3.33	(-10.20, 3.55)	0.343

^a^Intervention*time represents the status of the intervention group in comparison with the control group at six-month follow-up,

^b^Reference group,

^c^Sum of observations of baseline and follow-up data

^¶^ Gender, age, family size, and cluster effects were controlled for all the 15 schools

The intervention also did not show evidence of a significant effect on depression scores among adolescents. Depression symptoms did not change significantly over time and no significant difference was observed between the schools in intervention and control groups. (Table 4).

**Table 4:** GEE analyses; effect of intervention on depression symptom scores among adolescents^¶^.

	N = 2199^c^		
	*β*	(95% CI)	p-value
Intervention*Time^a^	0.30	(-0.21, 0.80)	0.249
**Time**			
Baseline^b^			
Month 6	-0.09	(-0.45, 0.27)	0.622
**School**			
Control^b^			
Intervention	0.20	(-1.10, 1.51)	0.760

^a^Intervention*time represents the status of the intervention group in comparison with the control group at six-month follow-up,

^b^Reference group,

^c^Sum of observations of baseline and follow-up data

^¶^ Gender, age, family size, and cluster effects were controlled for all the 15 schools

The intervention also did not show evidence of a significant effect on hope scores among adolescents. Hope scores did not change significantly over time and no significant difference was observed between the schools in intervention and control groups. (Table 5).

**Table 5:** GEE analyses; effect of intervention on hope among adolescents^¶^.

Variable	N = 2199^c^		
	*β*	(95% CI)	p-value
Intervention*Time^a^	-0.23	(-1.07, 0.61)	0.588
**Time**			
Baseline^b^			
Month 6	0.41	(-0.67, 0.99)	0.163
**School**			
Control^b^			
Intervention	-3.36	(-11.2, 4.52)	0.404

^a^Intervention*time represents the status of the intervention group in comparison with the control group at six-month follow-up,

^b^Reference group,

^c^Sum of observations of baseline and follow-up data

^¶^ Gender, age, family size, and cluster effects were controlled for all the 15 schools

## Discussion

The training of school teachers on psycho-social support did not improve mental health symptoms and hope among the adolescents from the intervention schools in Dhading, a district severely affected by the 2015 earthquake in Nepal. The findings of this study indicated that the intervention on psycho-social support training for the teachers alone may not have been adequate to address mental health in school settings after a disaster of massive scale.

We could not find the evidence that the intervention had an effect on PTSD symptoms (Table 3) and depression symptoms (Table 4) among the adolescents at a six-month follow-up. The finding is different from previous teacher-mediated interventions from China following a 2008 earthquake [25], Sri Lanka following a 2004 earthquake [28], and Turkey following a 1999 earthquake [26, 27]. The differences could be attributed to the time when the intervention was introduced and follow-up, the population studied, and sociocultural factors.

Moreover, PTSD and depression symptoms among the adolescents may not have improved in this study because the training was focused on teachers only. Although psycho-social support by teachers can help the adolescents with milder symptoms of PTSD and depression, severer symptoms would require specialized treatment [23]. The school-based interventions through health professionals such as psychologists or school nurses are known to yield better results for mental health outcomes [23]. The need of the health professionals to provide mental health and psycho-social support is undeniable. However, interventions requiring more specialized professionals would not be feasible in settings such as in Nepal with a severe shortage of mental health professionals with only 2 psychiatrists and 0.6 clinical psychologists per million population [39]. The training of mental health professionals and allocation of the health professionals in the schools across the country would require major policy reform which may take a very long time for a low-income and politically fragile country such as Nepal.

This study provides an evidence that teacher-mediated intervention may not be enough to improve PTSD and depression symptoms among the adolescents. The findings, however, could help in informing policy makers for the need of policy reforms to include health professionals in the schools. Meanwhile, teacher-mediated interventions could remain a more feasible and sustainable solution to address the adolescent mental health sooner. However, more focused and longer training might be necessary for the teachers to timely refer those with severer symptoms for further treatment.

PTSD and depression symptoms could also have persisted because of the massive scale of the disaster. Disasters can have a persistent negative psychological impact among 10% to 50% of the survivors [40]. PTSD symptoms tend to last for many years after the disaster [36]. Moreover, PTSD symptoms may not appear in a short time after a traumatic event and can manifest a long time later [41, 42]. Furthermore, new stressors for PTSD symptoms could have been introduced over time. The political instability in Nepal between 2015 and 2016 had led to delay in recovery process in earthquake-affected districts [43] which could also have affected the mental health of the adolescents. Further clinical examination would be necessary to confirm the actual severity of PTSD and depression symptoms.

This study also found no significant change in hope scores over time. Though no specific cut-off score for hope is available, the mean scores of hope in this study were around 22 at both baseline and follow-up for both intervention and control groups. The mean hope score in this study is higher than the mean score of around 13 in an intervention study conducted in Nepal in 2010 following a 10-year conflict in the affected districts [17]. Both the studies have used the same scale, and the finding from this study suggests that the level of hope among the adolescents affected by the 2015 earthquake is higher than the previous study. The nature of disasters could have attributed to the difference. Moreover, the country has transformed and undergone massive political and economic changes over the decade since the time of conflict which had ended in 2006.

Hope is considered to play a protective role for mental health [19]. It is a cognitive process directed towards belief to set and achieve goals [44]. In difficult circumstances such as earthquake, children and adolescents often look for role models and seek hope from adults [20]. Teachers can act as coaches to inspire and motivate adolescents to have a positive outlook towards the future despite the difficulties surrounding them [18]. The intervention in this study focused on training teachers to provide psycho-social support to the adolescents through a holistic approach in their everyday school activities [34]. Despite the lack of evidence on significant effect of the intervention on hope, the school teachers could still play important role in maintaining hope among the adolescents.

The findings of this study should be interpreted with four limitations. First, we used a self-administered questionnaire, which could have resulted in under- or over-reporting by the adolescents due to concerns about social desirability. Second, the results do not reflect the responses of the students with low attendance rate below 20% in the schools. The reasons for their absenteeism remains unknown. Third, the time interval between the intervention and follow-up is just six months, which could have been shorter to assess the actual change in mental health. Fourth, this study lacks information from additional sources such as guardians or parents which could have provided better perspectives. Despite the limitations, this study provides important information on what may work and what may not work in resource-limited and disaster-prone settings such as in Nepal.

## Conclusion

The findings of this study did not show significant effect of the intervention on improving mental health and hope. It indicated persistence of PTSD and depression symptoms among adolescents from a severely earthquake affected district highlighting the vulnerability of the adolescents following the disasters of massive scale. Moreover, the findings of this study indicated that the psycho-social support from the teachers alone may not help address PTSD and depression symptoms in school settings. It has policy implication for the need of including health professionals in the schools such as school nurses or psychologists to address the mental health problems. Nevertheless, the persistence of high levels of hope among the adolescents in this study suggests that training school teachers on psycho-social support is feasible and useful in low-resource and disaster-prone settings. Refresher training for the teachers could help continue the momentum of maintaining hope among adolescents. More focused and longer training is necessary to address PTSD and depression symptoms among adolescents affected by earthquake. Additionally, longer follow-up is required to assess the changes taking place over time.

## Supporting information

S1 File CONSORT Checklist. (DOC)

S2 File NHRC research proposal. (DOC)
