# Supplementary material for: Effect of psycho-social support by teachers on improving mental health and hope of adolescents in an earthquake-affected district in Nepal: A cluster randomized controlled trial
Source: PLoS One. 2019 Oct 1;14(10):e0223046. doi: 10.1371/journal.pone.0223046 (PMC6771999; doi:10.1371/journal.pone.0223046)
Supplement: S2 File — (DOC) [file pone.0223046.s002.doc]

# **S2 Research proposal**


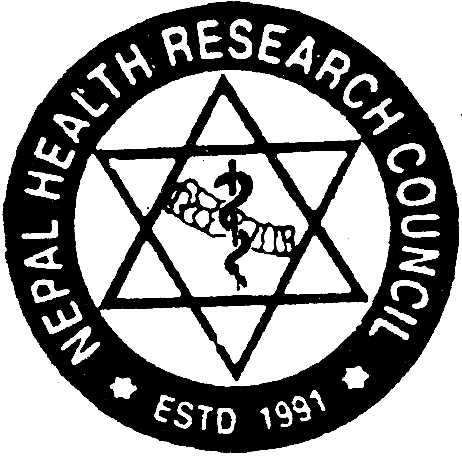


**Research Proposal Approval Format**

| **Research Title:** **Efficacy of psychosocial support training for school teachers to improve mental health and resilience of school going adolescents in district affected by earthquake in Nepal** |
| --- |

# **Nepal Health Research Council (NHRC)**

# **P.O. Box: 7626, Ramshah Path, Kathmandu, Nepal**

# **Tel:** +**977-1-4254220, 4227460, Fax:** +**977-1-4262469**

# **E-mail:** [**nhrc@nhrc.org.np**](mailto:nhrc@nhrc.org.np)**, Website:** [**http://www.nhrc.org.np**](http://www.nhrc.org.np/)

**Part III**

**Research Proposal Description**

1. Research Title:

| **Efficacy of psychosocial support training for school teachers to improve mental health and resilience of school going adolescents in district affected by earthquake in Nepal** |
| --- |

1. Proposal Summary (maximum 500 words):

| Adolescents are a vulnerable age group that face many mental health problems, which could become worse in the aftermath of disasters. On the other hand, this is also the age group that could cope better with resilience if proper psychosocial support is available. For school going adolescents, the best community based psychosocial support intervention could be provided through schools. The schoolteachers have a positive influence for children and adolescents in general. Teachers can provide timely and continued psychosocial support effectively to the children and adolescents faced by disaster and can also prepare them better for future unanticipated events.  For resource constraint and natural disaster prone settings like that of Nepal training of in service schoolteachers on psychosocial support for children and adolescents could be more sustainable. Though there have been psychosocial interventions immediately following earthquake in some worst hit districts of Nepal, the evidence of effectiveness in the long run remains inadequate. Moreover, the evidence on promoting resilience in disaster prone districts other than that affected by earthquake also remains inadequate.  This study aims to assess the efficacy of teacher’s training on adolescents’ mental health in a district severely affected by earthquake, and in a district least affected by earthquake in April, 2015 but is prone to disasters. The intervention for this in school comprise of psychosocial training for the schoolteachers in selected schools of the municipalities in Dhading and Myagdi.  Baseline study on the mental health status and resilience among the school going adolescents will first be assessed, followed by training of the schoolteachers on psychosocial support in intervention groups in both Dhading and Myagdi districts. This will be followed by follow up on the mental health status and resilience of the students at 3 months and 6 months follow up. During the follow up the level of knowledge decay among the teachers who received the training will also be assessed.  If the intervention proven to be effective, the same program can be expanded to other districts in Nepal. It can further be replicated to other disaster prone low-income countries. |
| --- |

1. Introduction:
   1. Background of Study (maximum 500 words):

| Disasters are a common global challenge that disrupts the community or a society leading to widespread human, material, economic and environmental losses.(1) It can take many lives and destroy homes or whole communities that can cause serious physical and psychological injuries.(2) The psychological injuries could lead to mental health problems like post traumatic stress disorder, anxiety disorder and depression.(3) Adolescents are vulnerable population that could be affected largely by mental health problems in the aftermath of disaster.(4)    The mental health problems manifested in adolescents could be more complex than in adults.(5) Responses to psychological trauma can be immediate or delayed following a disaster.(6) The symptoms may cover a wide range of behaviors, responses and severity. If not recognized and intervened timely, the consequences could be damaging and disabling in the long run.(7) In low income countries, compromised health system could add additional challenge to address these problems for this vulnerable population.(8)  School-based psychosocial support programs have shown to be effective to identify and reduce children’s disaster related trauma symptoms.(9, 10) Schools are one of the important community settings to reach a large number of children and adolescents.(11) A cluster controlled trial on psychosocial support for children affected by 10-year long civil war in Nepal showed the school based intervention to be effective.(9) Similar results were shown by a study conducted among children affected by political violence in Indonesia.(12)A systematic review on psychosocial support following armed conflicts in low and middle income countries suggested that school based interventions are feasible in resource limited settings.(2)  Moreover, schoolteachers have a positive influence for children and adolescents in general.( (11) Teachers can provide timely and continued psychosocial support effectively to the children and adolescents with mental health problems. (9) For disaster prone low-income settings, there could be additional factors such as poverty and delay in recovery that may influence the psychosocial status in the long run. Moreover, it has been suggested that psychosocial support should be continued and integrated according to the different phases of recovery.(13) However, studies on school based psychosocial interventions in the recovery phase from low-income countries affected by earthquake are still very limited  For resource constraint settings like that of Nepal training of in service schoolteachers on psychosocial support for children and adolescents could be more sustainable. This study aims to assess the efficacy of teacher’s training on adolescents’ mental health in a district severely affected by earthquake, and in a district least affected by earthquake in April, 2015 but is prone to disasters. |
| --- |

- 1. Statement of the Problem and Rationale / Justification (maximum 500 words)

| Nepal is a low income country that ranks 11^th^ globally in terms of earthquake risks.(14) The country faced a devastating earthquake of 7.8 Magnitude on April 25, 2015.(15) The earthquake killed over 8000 people and over 6000 were severely injured. The government also declared a state of emergency and called for international support.(16)  In addition to rescue and relief, around a dozen of local and international organizations provided psychological first aid to the earthquake victims. Some organizations also worked specifically for children. However, there were no standard protocols provided by the government for mental health and psychosocial support (MHPSS) for disaster settings. The mental health policies had not yet been implemented properly in the country. Thus, initial situational analysis suggests that most organizations faced challenges to provide proper MHPSS.(17)  Furthermore, all the schools in the earthquake affected districts remained closed for over a month following the earthquake. Thus, most schools were not directly involved in providing immediate psychosocial support to the school going children.  The damage caused by earthquake was further worsened by the political instability and shortage of essential supplies in the country. This has lead to delay in relief and reconstruction work in earthquake-affected areas. The immediate psychosocial problems following earthquake could have been compounded by the challenges caused by political instability. This could have worsened the mental health problems among many children and adolescents.  For low-income settings, there could be additional factors such as poverty and delay in recovery that may influence the psychosocial status in the long run. Moreover, it has been suggested that psychosocial support should be continued and integrated according to the different phases of recovery.(12)  Schools are one of the important community settings to reach a large number of children and adolescents. Schoolteachers have a positive influence for children and adolescents in general.(11)Teachers can provide timely and continued psychosocial support effectively to the children and adolescents with mental health problems. (18) However, studies on school based psychosocial interventions in the recovery phase from low-income countries affected by earthquake are still very limited.  There were some training programs for schoolteachers organized by various organizations in the affected districts. But most of them were conducted immediately after the earthquake and the evidence on effectiveness still remains unclear. Moreover, the effectiveness of psychosocial support for adolescents in general and as form of disaster preparedness remains unclear as well low-income countries like Nepal.  For resource constraint settings, training of in service schoolteachers on psychosocial support for children and adolescents could be more sustainable. Moreover, such intervention could provide evidence that could be applicable for future disasters in low-income settings. |
| --- |

- 1. Conceptual framework

The intervention in this study will focus around the principles that have been developed for psychosocial support in education. (19)

| Training teachers on 6 core principles of psychosocial support |
| --- |

Psychosocial support provided by teachers to adolescents that is

-Holistic

-Enabling

-Rights based

-Child centered

-Child friendly

-Comprehensive

Improved outcomes

- Reduced PTSD symptoms
- Reduced depression symptoms
- Increased Hope

School going adolescents affected by earthquake

- High PTSD symptoms
- High Depression
- Less Hope

Figure 1. Components of psychosocial support adapted from the training manual from UNWRA.

**Level of intervention at Layer 2**

Figure 2. Different layers of MHPSS intervention (Source: IASC, 2007)

- 1. Research Objectives / purpose / aim of the study:

General

| To assess the efficacy of teacher’s psychosocial training on mental health of school going adolescents |
| --- |

Specific

| - To assess the difference in the magnitude of mental health problems and resilience among school going adolescents between the districts severely affected and least affected by earthquake - To examine the determinants of mental health problems and resilience among school going adolescents in districts severely and slightly affected by earthquake - To examine the difference in efficacy of psychosocial support training of teachers on mental health status and resilience of school going adolescents in between severely affected and slightly affected by earthquake |
| --- |

1. Research Design and Methodology

Research Method

Qualitative Quantitative Combined

**√**

| **Baseline**  The baseline study will be mixed method in design. We will employ sequential explanatory method where the study will take place in two sequential phases. In the first phase, we will implement quantitative study, which will include data collection and analysis. We will then conduct a qualitative study in order to explain the initial quantitative results in depth.(19, 20) The two research methods will interact at the point where the quantitative results will guide the qualitative study.(21) The results from quantitative and qualitative study will be triangulated for the final interpretation.  Quantitative study: We will collect data from students of grade 6-8 to assess the mental health status and the determinants from the selected schools in Nilkantha and Beni municipalities. We will also assess the knowledge of the teachers from selected schools on psychosocial support for children.  Qualitative study: The aim of this study is to explain the findings of the quantitative study, which will also help us to improve our intervention. We will conduct eight focus group discussions with the schoolteachers in both Dhading and Myagdi districts.  **Intervention**  The intervention for this study will be provided to the schools selected under intervention group from both the districts. The intervention design is developed based on the suggestions from the summary report of the national consultative meeting held in Kathmandu, Nepal in October, 2015 on “Strengthening psycho-social support in post-disaster situation in schools and communities of rural Nepal”. The report highlighted the need of community based and sustainable interventions including the one in education. (22)  MHPSS interventions require a multilayered system to address the different needs in a more organized fashion.(23) According to Inter Agency Standing Committee (IASC), formal and informal education interventions fall under the second layer, namely community and family supports.(23) Thus, our intervention will focus on the second layer of the MHPSS interventions.(Figure 2) This layer of intervention focuses on a smaller number of people who are able to maintain their mental health and psychosocial wellbeing if they receive appropriate support system. The adolescents identified to have severe symptoms in this study will be referred to focused care (third layer) or to more specialized care (fourth layer) as per the results based on the baseline findings.  We will use already developed and validated curriculum for teachers training. We will conduct a four-day (a total of 16 hours) training on psychosocial support for the schoolteachers. We will adopt the standard training guidelines based on the United Nations Relief and Works Agency (UNRWA) on psychosocial support for education in emergencies. (24) The training will provide basic skills to the in service schoolteachers so that they will be able to support the psychosocial wellbeing of children affected. The training will comprise eight sessions in total with one to two hours for each session. The sessions will cover following areas:   1. Key concepts and principles of psychosocial support 2. How do children react to a crisis situation 3. The role of teachers in promoting psychosocial well-being 4. How to discuss a crisis with children 5. Activities for improved learning and recovery 6. How to manage challenging behavior in the classroom 7. Identifying and assisting children who may need more advanced support 8. Teachers’ well-being   We will select the experienced trainers working in the area of mental health and psychosocial support for children in this training.  **Follow up**  We will assess the mental health and psychosocial status of the same of cohorts of adolescents from the intervention and control groups at 3 months and 6 months intervals following the intervention. |
| --- |

Study variables:

*Socio-demographic variables*

The socio-demographic variables regarding the school children will be adopted from Nepal adolescents and youth survey.(25)The variables will include gender, date of birth, grade, ethnicity, address, living arrangement, parents’ education level, parents’ occupation and distance between home to school.

*Outcome variables*

The outcome variables of this study will be mental health status and resilience among the adolescents.

Mental health status:

For mental health status we will assess child posttraumatic stress disorder (PTSD) symptoms and self reported depression symptoms.

- Post Traumatic Stress Disorder symptoms: We will use child PTSD symptom scale (CPSS), which has already been validated in Nepal. The CPSS has 17 items that correspond to PTSD diagnostic criteria in the Diagnostic and Statistical Manual of Mental Disorders (DSM-IV). It consists of 4-point scale from 0 to 51. Higher scores indicate more likely to be symptomatic for PTSD.(26)
- Self reported depression symptoms: The Depression Self Rating Scale (DSRS) is an 18-item self-report measure for children, which has been used in a range of cross-cultural contexts. This instrument records symptoms over the past week. Items are presented as statements, e.g. “I sleep very well.” Responses are a 0 ‘mostly’, 1 ‘sometimes’, 2 ‘never’.(26)

Resilience:

Resilience is defined as "the process of, capacity for, or outcome of successful adaptation despite challenging or threatening circumstances" (27) In a previous study conducted among adolescents in Nepal, positive aspects of wellbeing were used as antecedents of resilience. (28) We will use the same scales to assess resilience in our study, as these scales have been considered to be suitable in the context of Nepal. We will use two scales namely Children’s Hope Scale (CHS) and Concern for Others Scale.

- CHS assesses a sense of hope, with a higher score denoting more hope.
- Concern for Others Scale measures the child’s feeling of concern for, and desire to help, other people with higher scores reflecting more pro-social behavior.(29)

*Independent variables*

Health related quality of life:

We will consider health related quality of life (HRQOL) of children and adolescents as an independent variable. We will use the Kinder LebensqualitätFragebogen (KINDL^R^) to assess HRQOL of the adolescents. The scale covers “Physical Well-Being”, “Emotional Well-Being”, “Self-Esteem”, “Family”, “Friends”, and “School”.(30)

Knowledge of the teachers on psychosocial support:

We will use the questionnaires from the training manual on psychosocial support for education in emergencies. There are 8 sections that cover different areas of psychosocial support in education for children with true or false questions for one each for 8 sessions of training. (24)

Study Variables:

Type of Study (Specify):

Study Site and Its Justification:

| We will include two districts for this study: Dhading, one of the worst affected districts by earthquake in April, 2015 and Myagdi for comparison as one of the least affected district. Both the districts lie in hilly region and are considered remote districts of Nepal.  We will select the secondary and lower secondary government schools from Nilkantha municipality in Dhading and Beni municipality from Myagdi. |
| --- |

Study Population (Specify):

| The study population for this study will be school going adolescents. We chose early adolescents from grade 6 to 8 who would in an average fall into the age group of 11 to 14 years in this study. This is the beginning of adolescence period where timely psychosocial support could help in the long run throughout their adolescence period. This is the beginning of transition period where along with physical changes they also go through new emotional challenges as well. On the other hand, it is also a time when they are more receptive, more resilient as compared to older aodlescents. Moreover, the validated questionnaires that will be used in this study are age specific and the ones validated in Nepal have been considred to be suitable for this specific age group.  The intervention is on the schoolteachers from schools in the intervention groups in both the districts. |
| --- |

Study Unit:

| Lower secondary and secondary schools of the municipalities of the selected districts |
| --- |

Sampling Methods / Techniques (Specify):

The municipalities will be selected purposively. The schools in each municipality will be selected randomly and randomly allocated to intervention and control arm. Equal number of male and female students will be randomly selected from each school between the grades 6 to 8.

.

Sample size (with justification):

The sample size was calculated based on the mean values of Child PTSD Symptom Scale (CPSS) from a similar intervention study conducted in Nepal [9]. The minimum required sample size for this study was calculated to be 498 for each group with a power of 80% and 5% level of significance.

However, considering the incomplete questionnaires into account, we will extrapolate the sample size and include 800 students in each group from Dhading district. There are lesser schools in Myagdi district, so we will take 400 students for each arm in Myagdi district. Thus, we will have 2400 students in total with 1200 each in intervention and control groups.

The schoolteachers will be selected purposively based on the number of teachers appointed in each school and their willingness to participate. We will select at least 20 teachers with 5 teachers from each school from the intervention group for training in each district.

| **Schools**:  We will include government schools having lower secondary level from grade 6 to 8 in this study. The schools not running at the time of study will be excluded.  **Students:**  We will include the students enrolled in the selected schools from grade 6 to 8 considering the fact the average age group in these grades would fall under aged 11 to 14 years. The students who were found to have diagnosed psychiatric problems will be excluded from this study, but will be assured that they have received specialized care. The students with severe mental disability who are unable to respond to the questions will also be excluded from the study, but will be referred to specialized care. The students with severe PTSD and depression symptoms identified through baseline study will also be referred to the specialized psychiatric care. The specialized care center identified for referral is Department of Psychiatry, Tribhuwan University Teaching Hospital, Kathmandu, Nepal.  **Teachers**:  We will include in service schoolteachers teaching lower secondary and secondary level from the selected schools. The part time teachers and those who plan to leave or will be transferred from the selected schools within intervention period will be excluded from the intervention. However, they will be considered eligible to participate in the focus group discussions. |
| --- |

Criteria for Sample Selection:

Data Collection Technique / Methods (Specify):

| After being informed of all the details of the study through information sheet for the guardians, teachers,  and students, we will obtain the written informed consent from them all. The teachers who are willing to participate in the training will provide us written informed consent to be part of the study for the next six months. Likewise, the students willing to participate will provide us written informed consent form from their guardian and themselves.  Considering the sensitive nature of the study and the emotions involved, the students may not open up easiliy and answer to the questions asked by the interviewer. To reduce the risk of social desirability bias, the data collection will be self-administered. However, the research assistants will assist the students in filling out the forms in a classroom. The research assistants will also be available on the spot to resolve any problem or explain the questions to the students, when they are confused. The tools used are considered suitable for the age group of 11 to 14 years of age, thus will be considered suitable for self-administration.  The knowledge of teachers will also collected through self-administered questionnaire.  The focus group discussions for the teachers will be conducted through focus group discussion checklists. The checklists will be developed based on the baseline quantitative study results. |
| --- |

Validity and Reliability of the Study Tools:

All the tools used in this study are the tools already validated or translated, used and published in Nepal considered suitable to be used for the adolescents

Potential Biases (if applicable):

As the study population is students and questions asked are on mental health, considering the taboos related to mental health in our society, the responses could lead to social desirability bias.

However we are using standard validated tools specifically meant for adolescents so the chances of bias is minimal.

Limitation of the Study:

This study will be conducted in the municipalities of the selected districts, thus may not represent the mental health status of the school going adolescents from the more remote parts of the same district or the whole country.

1. Plan for Supervision and Monitoring:

The study will be conducted in three phases, the baseline study will be conducted prior to intervention, with follow ups at 3 and 6 months after the intervention. The research assistants hired for the study will be carefully orientated trained on data collection. The researcher will have a discussion with research assistants at the end of every working day. Any kind of misunderstanding and mistakes during conducting will be cleared. Informed consent process will be closely monitored.

The intervention of the study is psychosocial support training for the teachers, which will be carefully conducted by the trained professionals.

To monitor the knowledge decay among the teachers who received the training, we will be assessing their knowledge on psychosocial support in education during each follow up visit.

The mental health status and resilience of the students will also be monitored through data collection at the Follow up visits.

.

1. Plan for Data Management and Analysis:

All the information collected will remain strictly confidential. The names of the participants will not be recorded, instead a code number will be given to each participant. The collected questionnaire will be stored in a locked cabinet with the PI at the Department of community health sciences, the University of Tokyo.

The collected data will be carefully entered into Epi info and then transferred to SPSS.

For quantitative analysis, we will conduct both descriptive and multivariate analysis. For descriptive analysis we will conduct chi square test and independent sample T-test to assess the difference between two groups. We will also conduct multivariate logistic regression analysis.

We will conduct GEE to assess differences in the psychosocial status between the groups and, before and after the intervention.

For qualitative analysis, we will use the thematic approach. We will organize the information into themes first. We will use some quotes that reflected the original reaction from the participants and present in their own words. This will give more insight into the original perceptions.

Expected Outcome of the Research:

This research hypothesized that the teachers training on psychosocial support will be an effective intervention for improving mental health and resilience among school gong adolescents.

1. Plan for Dissemination of Research Results:

The results of this study will be disseminated through PhD thesis, publications in peer reviewed journals and if possible through national and international conferences.

1. Plan for Utilization of the Research Findings (optional):

The initial results will be shared with concerned mental health and psychosocial support organizations. It will also be published as an academic paper.

1. Work Plan *(should include duration of study, tentative date of starting the project and work schedule / Gantt chart):*

| **Activities** |  | | | | | | | | | | | | |  | | | |
| --- | --- | --- | --- | --- | --- | --- | --- | --- | --- | --- | --- | --- | --- | --- | --- | --- | --- |
|  | **2016** | | | | | | | | | | | | |  | **2017** | | |
|  | **12** | **1** | **2** | **3** | **4** | **5** | **6** | **7** | **8** | **9** | **10** | **11** | **12** | **1** | | **2** | **3** |
| Finalize proposal |  |  |  |  |  |  |  |  |  |  |  |  |  |  | |  |  |
| Ethical approval from University of Tokyo |  |  |  |  |  |  |  |  |  |  |  |  |  |  | |  |  |
| Submission of proposal to NHRC |  |  |  |  |  |  |  |  |  |  |  |  |  |  | |  |  |
| Data collection (Baseline) |  |  |  |  |  |  |  |  |  |  |  |  |  |  | |  |  |
| Data collection, entry, cleaning and analysis (Baseline |  |  |  |  |  |  |  |  |  |  |  |  |  |  | |  |  |
| Paper writing for baseline data/ Review paper |  |  |  |  |  |  |  |  |  |  |  |  |  |  | |  |  |
| 1^st^ Follow up data collection |  |  |  |  |  |  |  |  |  |  |  |  |  |  | |  |  |
| Data entry, analysis |  |  |  |  |  |  |  |  |  |  |  |  |  |  | |  |  |
| 2^nd^ Follow up data collection |  |  |  |  |  |  |  |  |  |  |  |  |  |  | |  |  |
| Data collection, entry, cleaning and analysis (Baseline |  |  |  |  |  |  |  |  |  |  |  |  |  |  | |  |  |
| Thesis writing |  |  |  |  |  |  |  |  |  |  |  |  |  |  | |  |  |

**Part – IV**

**Ethical Consideration**

22. Regarding the human participants:

Are human participants required in this research? If yes, provide justification.

Yes (*provide justification*) No

√

This is the study to be conducted going adolescents’ mental health status

How many participants are required for the research? Explain.

| Based on the sample size calculation the total number of participants required are 2400 students, with 1200 each on intervention and control arm. |
| --- |

What is the frequency of the participant’s involvement in the research? Explain.

| The participants will be involved three times in the study, at the baseline, at three months follow up and six months follow up. |
| --- |

Clearly indicate the participant's responsibilities in the research. What is expected of the research participants during the research?

| The research participants are required to answer the self-reported questionnaire through face-to-face interview. The questions asked are age specific for the age group of 11- 14 years old (grade 6 to 8)  The teachers willing to participate in the trainings will also be assessed three times, at the baseline and at three months and six months follow up. |
| --- |

Are vulnerable members of the population required for this research? If yes, provide justification.

| The research participants of this study will be adolescents from grade 6 to 8. We will first obtain written informed consent from the guardians/caretakers currently living with the adolescents to collect the data from the students. We will then obtain the written informed consent from the students as well. |
| --- |

Are there any risks involved for the participants? If yes, identify clearly what are the expected risks for the human participants in the research and provide a justification for these risks.

| No, there are no risks involved to the participants. The adolescents identified through the research having moderate to severe mental health problems will be referred to the specialized mental health care. |
| --- |

Are there any benefits involved for the participants? If yes, identify clearly what are the expected benefits for the participants.

| This study will also help as a school based screening of mental health for the adolescents.  There will be basic incentive such daily allowance to cover the travel cost and food for the teachers who participate for the training. |
| --- |

23. Informed Consent Form / Ethical Issues:

Statements required in the Informed Consent Form include:

A statement that the human participants can withdraw from the study at any time without giving reason and without fear. State clearly how the participants can opt out the study.

A statement guaranteeing the confidentiality of the research participants.

If required, a statement on any compensation that might be given to the research participant and or their community.

A statement indicating that the participants has understood all the information in the consent form and is willing to volunteer / participate in the research.

Signature space for the research participants, a witness, and the date.

*(Informed Consent form should be submitted in English and in the language appropriate to the research participants)*

Obtaining the Consent

How informed consent is obtained from the research participants?

Verbal Written

√

Please indicate who is responsible for obtaining informed consent from the participants in this research study?

Rolina Dhital is responsible for obtaining informed consent from the participants

…………………………………………..………………………………

Is there anything being withheld from the research participants at the time the informed consent is being sought?

If yes, explain ……………………………………………………………………………

Is the research sensitive to the Nepali culture and the social values?

Yes No Explain.

√

……………………………………………………………………………

Is health insurance *(if applicable)* being made available to the research participants? If yes, please provide the necessary insurance data.

……………………………………………………………………………

(Include in consent form)

**Appendix 1**

**References**

1. Gerdin M, Clarke M, Allen C, Kayabu B, Summerskill W, Devane D, et al. Optimal evidence in difficult settings: improving health interventions and decision making in disasters. PLoS Med. 2014;11(4):e1001632.

2. Norris FH, Friedman MJ, Watson PJ, Byrne CM, Diaz E, Kaniasty K. 60,000 disaster victims speak: Part I. An empirical review of the empirical literature, 1981-2001. Psychiatry. 2002;65(3):207-39.

3. Guo J, Wang X, Yuan J, Zhang W, Tian D, Qu Z. The symptoms of posttraumatic stress disorder and depression among adult earthquake survivors in China. J Nerv Ment Dis. 2015;203(6):469-72.

4. Fujiwara T, Yagi J, Homma H, Mashiko H, Nagao K, Okuyama M, et al. Clinically significant behavior problems among young children 2 years after the Great East Japan Earthquake. PLoS One. 2014;9(10):e109342.

5. Olteanu A, Arnberger R, Grant R, Davis C, Abramson D, Asola J. Persistence of mental health needs among children affected by Hurricane Katrina in New Orleans. Prehosp Disaster Med. 2011;26(1):3-6.

6. Jensen TK, Ellestad A, Dyb G. Children and adolescents' self-reported coping strategies during the Southeast Asian Tsunami. Br J Clin Psychol. 2013;52(1):92-106.

7. Jacobs MB, Harville EW. Long-Term Mental Health Among Low-Income, Minority Women Following Exposure to Multiple Natural Disasters in Early and Late Adolescence Compared to Adulthood. Child Youth Care Forum. 2015;44(4):511-25.

8. Barry MM, Clarke AM, Jenkins R, Patel V. A systematic review of the effectiveness of mental health promotion interventions for young people in low and middle income countries. BMC Public Health. 2013;13:835.

9. Jordans MJ, Komproe IH, Tol WA, Kohrt BA, Luitel NP, Macy RD, et al. Evaluation of a classroom-based psychosocial intervention in conflict-affected Nepal: a cluster randomized controlled trial. J Child Psychol Psychiatry. 2010;51(7):818-26.

10. Chemtob CM, Nakashima JP, Hamada RS. Psychosocial intervention for postdisaster trauma symptoms in elementary school children: a controlled community field study. Arch Pediatr Adolesc Med. 2002;156(3):211-6.

11. Weare K, Nind M. Mental health promotion and problem prevention in schools: what does the evidence say? Health Promot Int. 2011;26 Suppl 1:i29-69.

12. Tol WA, Song S, Jordans MJ. Annual Research Review: Resilience and mental health in children and adolescents living in areas of armed conflict--a systematic review of findings in low- and middle-income countries. J Child Psychol Psychiatry. 2013;54(4):445-60.

13. Rao K. Psychosocial support in disaster-affected communities. Int Rev Psychiatry. 2006;18(6):501-5.

14. UNDP. ISDR Global Assessment Report on Poverty and Disaster Risk 2009, global assessment of risk, Nepal Country report. Available at: Accessed June 11, 2015. Kathmandu, Nepal.

15. Government of Nepal MoHA. Nepal Disaster Risk Reduction Portal, Kathmandu. Nepal earthquake 2071; situation update as of 11th May.

16. Nepal Go. Nepal earthquake 2015 Post disaster needs assessment. National Planning Commission, Government of Nepal; 2015.

17. Corps IM. Rapid mental health and psychosocial support needs assessment: services, identified needs and recommendations following April and May 2015 earthquakes in Nepal. Washington DC, US: International Medical Corps; 2015.

18. Tol WA, Komproe IH, Susanty D, Jordans MJ, Macy RD, De Jong JT. School-based mental health intervention for children affected by political violence in Indonesia: a cluster randomized trial. JAMA. 2008;300(6):655-62.

19. Creswell JW CP. Designing and conducting mixed methods research. 2nd edition ed: Thousand Oaks: SAGE Publications; 2010.

20. Brenner S, Muula AS, Robyn PJ, Bärnighausen T, Sarker M, Mathanga DP, et al. Design of an impact evaluation using a mixed methods model--an explanatory assessment of the effects of results-based financing mechanisms on maternal healthcare services in Malawi. BMC Health Serv Res. 2014;14:180.

21. Creswell JW CP. Choosing a mixed method design 2nd edition ed. Califormia, US: Thousand Oaks: SAGE Publications; 2010.

22. Green Tara Nepal and The University of Tokyo. Strengthening psycho-social support in post-disaster situation in schools and communities of rural Nepal. Green Tara Nepal, The University of Tokyo. Kathmandu; 2015.

23. Inter Agency Standing Committee. IASC guidelines on mental health and psychosocial support in emergency settings. Geneva: IASC; 2007

24. UNRWA. Psychosocial support for education in emergencies - training and resource package for teachers and counsellors. United Nations Relief Works Agency. Gaza: United Nations Relief Works Agency; 2013.

25.Government of Nepal, Ministry of Health and Population. Adolescent and youth survey 2010/11. Kathamndu, Nepal: Ministry of Health and Population; 2012.

26. Kohrt BA, Jordans MJ, Tol WA, Luitel NP, Maharjan SM, Upadhaya N. Validation of cross-cultural child mental health and psychosocial research instruments: adapting the Depression Self-Rating Scale and Child PTSD Symptom Scale in Nepal. BMC Psychiatry. 2011;11(1):127.

27. Yamaguchi N, Poudel KC, Jimba M. Health-related quality of life, depression, and self-esteem in adolescents with leprosy-affected parents: results of a cross-sectional study in Nepal. BMC Public Health. 2013;13:22.
